# Aging in High-Risk Coastal Regions: Examining Social Infrastructure Needs of Older Adults Living in the Gulf Coast

**DOI:** 10.1093/geroni/igaa057.1980

**Published:** 2020-12-16

**Authors:** Alexis Merdjanoff

**Affiliations:** New York University, New York, New York, United States

## Abstract

This study examines how coastal erosion, flooding susceptibility, and extreme hazard risk in Louisiana communities shape decisions to age in place. The decision to age in place is not only related to one’s physical health and cognitive capabilities but strongly connected to neighborhood cohesion, sociocultural contexts, economic resources, familiarity with surroundings, and a sense of security. However, research on the types of individual and community resources that older adults need in order to successfully age in environmentally vulnerable communities is exceptionally sparse. Using in-depth interview data collected from older adults (n=20) living in coastal Louisiana parishes, this research aims: 1) to gain a deeper understanding of how coastal erosion and frequent flooding influence the decision to age in place; 2) to compile evidence as to how coastal communities can create resources that promote resilience, despite environmental risk; and 3) to use this evidence to increase awareness and enhance policy discussions on coastal adaptation.

